# Microbiota of Breast Tissue and Its Potential Association with Regional Recurrence of Breast Cancer in Korean Women

**DOI:** 10.4014/jmb.2106.06039

**Published:** 2021-09-25

**Authors:** Hyo-Eun Kim, Jongjin Kim, Sejung Maeng, Bumjo Oh, Ki-Tae Hwang, Bong-Soo Kim

**Affiliations:** 1 Department of Life Science, Multidisciplinary Genome Institute, Hallym University, Chuncheon, Gangwon-do 24252, Republic of Korea; 2 Department of Surgery, Seoul Metropolitan Government Seoul National University Boramae Medical Center, Seoul 07061, Republic of Korea; 3 Department of Family Medicine, Seoul Metropolitan Government Seoul National University Boramae Medical Center, Seoul 07061, Republic of Korea; 4 The Korean Institute of Nutrition, Hallym University, Chuncheon, Gangwon-do 24252, Republic of Korea

**Keywords:** Breast cancer, tissue microbiota, regional recurrence, microbiota cluster, Enterococcus

## Abstract

Recent studies have reported dysbiosis of the microbiome in breast tissue collected from patients with breast cancer and the association between the microbiota and disease progression. However, the role of the microbiota in breast tissue remains unclear, possibly due to the complexity of breast cancer and various factors, including racial and geographical differences, influencing microbiota in breast tissue. Here, to determine the potential role of microbiota in breast tumor tissue, we analyzed 141 tissue samples based on three different tissue types (tumor, adjacent normal, and lymph node tissues) from the same patients with breast cancer in Korea. The microbiota was not simply distinguishable based on tissue types. However, the microbiota could be divided into two cluster types, even within the same tissue type, and the clinicopathologic factors were differently correlated in the two cluster types. Risk of regional recurrence was also significantly different between the microbiota cluster types (*p* = 0.014). In predicted function analysis, the pentose and glucuronate interconversions were significantly different between the cluster types (*q* < 0.001), and *Enterococcus* was the main genus contributing to these differences (*q* < 0.01). Results showed that the microbiota of breast tissue could interact with the host and influence the risk of regional recurrence. Although further studies would be recommended to validate our results, this study could expand our understanding on the breast tissue microbiota, and the results might be applied to develop novel prediction methods and treatments for patients with breast cancer.

## Introduction

Breast cancer is one of the three most common cancers in women, and its incidence has been increasing worldwide [1]. Although extensive studies have been conducted, the etiology of breast cancer remains elusive. Genetic risk factors, such as *BRCA1/2* mutations, and environmental factors, such as lifestyle, obesity, alcohol, and diet, have been identified as related to breast cancer. Nevertheless, these factors could only explain the global burden of this disease to a limited extent [2]. Several studies have shown that an imbalance in estrogen hormone levels plays an important role in carcinogenesis, thus suggesting that it is a major risk factor [3, 4]. Recently, the human microbiome has attracted much attention as an additional risk factor, since the microbiome in the body plays a significant role in human health and diseases [5, 6]. Associations between the microbiota and the development and aggressiveness of cancer have been reported for various cancers [7-10]. For breast cancer, differences in the gut microbiome between patients with breast cancer and healthy subjects have been reported previously, and the involvement of the gut microbiome in estrogen metabolism has been implied [11-13]. However, only a few studies have characterized the microbiota in breast tumor tissue, and its role in breast cancer remains to be unraveled.

Breast tissue was previously thought to be sterile; however, it is now known to contain a diverse and unique microbiota [14]. Several studies have confirmed the presence of microbiota in breast tissue, with unique results that are distinct across pathologically normal, benign, and malignant breast tissue [12, 15-19]. The microbiota in nipple aspirate fluid and sentinel lymph nodes has also been characterized for patients with breast cancer [20, 21]. Recently, the microbiome of breast cancer tumors has been reported to be rich and diverse, compared to that of other cancers [22]. Breast tissue includes widespread vasculature, lymphatics, and diffusely located lobules and ducts leading from the nipple and offering a nutrient-rich environment, including fatty acids, which is conducive to the growth of unique microbiota. A potential origin of the microbiota in breast tissue occurs via translocation from the gut, skin, through the nipple-areolar orifices, and nipple-oral contact during either lactation or sexual contact [18]. Since the microenvironment of cancer includes tumor cells, immune cells, and microbiota, microbiota dysbiosis could influence tumor growth, inflammation-mediated carcinogenesis, and immune invasion [23, 24]. However, investigations of the microbiome in breast tissue remain insufficient and the specific microbial signature responsible for breast carcinogenesis is still unclear. Comparative studies of microbiota between breast tumor tissue and adjacent normal tissue in patients are limited [17, 19]. Further, geographic and racial differences in breast tissue microbiome have been reported [17, 25], and such differences were mainly attributed to environmental factors, such as diet, habits, and lifestyle [26]. Therefore, comparative analyses of microbiota between breast tumor tissue and adjacent tissue, obtained from Korean women, and their potential roles in the progression of breast cancer, are necessary to extend our understanding of the tissue microbiota in breast cancer.

This study aimed to determine differences in the microbiota according to tissue types in patients with breast cancer and their correlation with clinical features and the progression of cancer. We compared the microbiota across breast tumor tissue, adjacent normal tissue, and lymph node tissue from Korean patients with breast cancer. The correlations between microbiota and clinical features were analyzed, and the potential roles of microbiota were determined. This study highlights the fact that different microbiota in breast tissue can influence regional recurrence and are associated with clinical features.

## Materials and Methods

### Study Subjects and Sample Collection

We have prospectively collected breast tissues such as primary breast tumor tissues, normal breast tissues, or metastatic lymph node tissues from 218 female patients with primary invasive breast cancer and axillar lymph node metastases, who underwent total mastectomy at Seoul Metropolitan Government Seoul National University Boramae Medical Center since 2006. Among them, all of three different kinds of tissue samples (normal, tumor, lymph node tissues) were available from 47 patients and we enrolled them in this study. A total of 141 tissue samples (47 normal, 47 tumor, and 47 lymph node tissues) were simultaneously obtained from the same patient in the operation room during breast cancer surgery. Normal tissue samples were obtained from surgical specimens, sufficiently away from cancer tissues. Tumor tissue samples were obtained from the cores of tumor tissues without any contamination of normal tissues. Lymph node samples were also obtained from the cores of metastatic axillar lymph nodes, and we ensured that every node sample was collected from a metastatic node, confirmed by a pathologist using a frozen section. All processes of tissue sampling were conducted under aseptic conditions in the operation room. To check microbial contamination in the operation room, an empty cryotube containing phosphate-buffered saline was prepared in the room. The cap of tube was left open near the sampling tubes during the operation, and closed when the operation ended. Five randomly selected contamination controls were also analyzed along with tissue samples. The collected samples were frozen at −80°C until further processing.

This study was approved by the Institutional Review Board of Seoul Metropolitan Government Seoul National University Boramae Medical Center (No. 16-2016-146) and performed in accordance with the Declaration of Helsinki. Written informed consent was obtained from all participants under institutional review board-approval protocols.

### Clinicopathologic Parameters

Age was defined as the age at diagnosis of primary breast cancer. TNM staging was determined according to the 8^th^ edition of the American Joint Committee on Cancer. Status of estrogen receptor (ER) or progesterone receptor (PgR) was defined based on the results of immunohistochemistry tests [27]. Hormone receptor (HR) status was defined as positive when the result of immunohistochemistry for either ER or PgR was positive. HR status was defined as negative when results for both ER and PgR were negative. Human epidermal growth factor receptor 2 (HER2) was defined as negative when the immunohistochemistry results were negative or 1+ and as positive when the results were 3+; when the results were 2+, the positivity of HER2 was defined according to the results of fluorescence *in situ* hybridization [28]. Histologic grade (HG) and nuclear grade (NG) were defined according to the modified Scarff-Bloom-Richardson grading system. Lymphovascular invasion was defined as positive when either lymphatic invasion or vascular invasion was positive. Body mass index was defined as the ratio of body weight (in kilograms) to height (in square meters). Operations were classified as lumpectomy or mastectomy according to the extent of surgery in the breast tissue.

### DNA Extraction and Bacterial 16S rRNA Gene Sequencing

Metagenomic DNA was extracted from 0.25 g of each tissue using a RNeasy Power Microbiome kit (Qiagen, Germany). The bacterial 16S rRNA gene (targeted V1–V3 region) was amplified based on the protocol for preparing a 16S metagenomics sequencing library with the MiSeq system (Illumina, Inc., USA) described previously [29], and the amplification conditions were modified for tissue microbiota according to a previous study [30]. Recent studies reported that the V1–V3 region could show high resolution in identification of taxonomic composition and more actual abundance of bacteria than other variable regions [31, 32], thus we used this target region. The first step of amplification was performed in a final volume of 50 μl containing 1 μM of each primer, 2.5 U Ex Taq polymerase (Takara Bio, Japan), 5 μl of 10 × Ex Taq buffer, 4 μl dNTP mixture, and 2 μl template DNA. The amplification consisted of initial denaturation at 94°C for 10 min, followed by 35 cycles of denaturation at 94°C for 1 min, annealing at 68°C for 1 min, extension at 72°C for 1 min, and a final extension at 72°C for 10 min [30]. The purification and size selection were performed using Agencourt AMPure XP beads (Beckman Coulter, USA). Index PCR was performed using 5 μl of purified PCR product in a final volume of 50 μl using the Nextera XT index kit (Illumina). The index PCR conditions consisted of initial denaturation at 94°C for 10 min, followed by 12 cycles of denaturation at 94°C for 1 min, annealing at 65°C for 1 min, extension at 72°C for 1 min, and a final extension at 72°C for 10 min. The amplicons of each sample were purified again using Agencourt AMPure XP beads (Beckman Coulter). The concentration of each library was determined using the Takara PCR Thermal Cycler Dice Real Time System III with the GenNext NGS Library Quantification Kit (Toyobo, Japan). Negative controls were included at every step to check contamination, and 8 negative controls (5 of empty cryotube for check operation room, 1 of DNA-free water added to the DNA extraction kit and 1 of the DNA clean-up kit, and 1 of the library preparation with DNA-free water) were sequenced with samples for quality control of sequencing. Equimolar concentrations of each library from different samples were pooled and sequenced on the Illumina Miseq system (300-bp paired ends) according to the manufacturer’s instructions.

### Estimation of Bacterial Amounts Using Quantitative Real-time PCR

The total amount of bacteria in each sample was measured and compared by quantitative real-time PCR based on the 16S rRNA gene, as described previously [33, 34]. The amplification was performed using 340F (5′-TCCTACGGGAGGCAGCAG-3′) and 518R (5′-ATTACCGCGGCTGCTGG-3′) primers on a Thermal Cycler Dice Real Time System III (Takara Bio). Amplification reactions were carried out for each sample in a final volume of 25 μl, containing 12.5 μl of 2 × SYBR green premix Ex Taq (Takara Bio), 2 μM of each primer, and 1 μl of DNA template (10-fold serial dilution of DNA) or distilled water (negative control). The reaction conditions consisted of initial denaturation at 95°C for 5 min, followed by 40 cycles of 95°C for 5 s and 60°C for 30 s. Standard curves were generated from parallel reactions with serial log-concentrations of the copy number of bacterial 16S rRNA gene from *Escherichia coli* K12 W3110, and each reaction was performed in triplicate. The calculated rRNA gene copy numbers were divided by 4.2 (the average copy number of the 16S rRNA gene in bacteria) to estimate the total cell number [35].

### Sequencing Data Analysis

Raw sequence reads were analyzed using CLC genomics workbench v.11.0.1 with the Microbial Genomics Module (Qiagen) as described previously [29, 34]. Briefly, raw sequences were merged, and sequences with short read-lengths (< 430 bp of merged reads) or low-quality score (Q < 25), along with primer sequences, were removed using the USEARCH pipeline v.10.0.240 (http://www.drive5.com/usearch). Chimeric sequences were detected and removed using the UPARSE tool [36]. Sequences were then clustered into operational taxonomic units (OTUs) based on 97% identity with the EzTaxon-e database [37], and representative sequences in each OTUs cluster were identified based on the EzTaxon-e database. Sequence reads detected in negative controls were removed from sequences in tissue samples. To compare diversity indices across samples, read numbers in each sample were normalized by random subsampling and the indices were calculated by MOTHUR [38].

Clustering of microbiota in tumor tissue was performed to evaluate the differences in microbiota within the same tissue. The clustering was conducted by enterotype analysis using the 'Cluster' and 'ClusterSim' packages, based on the Jensen-Shannon divergence metrics, and visualization of the cluster was conducted using 'ade4' package in R software (https://enterotype.embl.de/). The microbiota in normal and lymph node tissues was analyzed based on the cluster of tumor tissue microbiota. The significant differential taxa between clusters were determined using the Linear Discriminant Analysis Effect Size (LEfSe) algorithm [39]. The correlation of different genera between clusters with clinical features was analyzed by the Spearman’s correlation and visualized using a heatmap with R software ver. 3.5.1. The function of microbiota was predicted using PICRUSt2 (https://github.com/picrust/picrust2), and the predicted functions were analyzed based on Kyoto Encyclopedia of Genes and Genomics (KEGG) pathways.

### Statistical Analyses

A two-sample *t*-test was used to determine the difference in mean age, and Pearson’s χ^2^ test was used to determine differences in clinicopathologic characteristics across groups. We analyzed the overall survival and disease-free survival, defining the time intervals for each as the time from operation to death due to any cause, and that from operation to recurrence of any type, respectively. Breast cancer recurrence types included local recurrence, regional recurrence, and systemic recurrence. Loco-regional recurrence was also calculated in this study. This study had no case with contralateral breast cancer. The Kaplan–Meier estimator was used to analyze survival rates. The log-rank test was used to determine the significance of differences between two or more survival curves. All statistical analyses for clinical features were carried out using IBM SPSS Statistics, version 20.0 (IBM Corp., USA). All tests were two-sided and statistical significance was considered when the *p* value was less than 0.05.

Differences in microbiota and measured bacterial amounts across samples, as well as the significance of correlations between microbes and clinical features, were analyzed using the Mann-Whitney U-test and Kruskal-Wallis test in R software. Differences in beta-diversity were visualized using non-metric multidimensional scaling (NMDS) plots and tested for inference by permutation multivariate analysis of variance (PERMANOVA) with 999 permutations based on the Bray-Curtis distance. Statistical tests of predicted functions were performed using STAMP [40]. Significantly different predicted KEGG pathways were determined using the Kruskal-Wallis H-test and Welch’s t-test with Benjamini-Hochberg false discovery rate (FDR) correction (*q* value), and a post-hoc test was performed using the Tukey-Kramer method. Results with *p* values or *q* values less than 0.05 were considered statistically significant.

### Data Availability

The sequence reads obtained from this study are available in the EMBL SRA database under the project number PRJEB37724 (https://ebi.ac.uk/ena/browser/view/PRJEB37724).

## Results 

### Clinicopathologic Characteristics of Subjects

Clinicopathologic characteristics of analyzed patients are presented in Table 1. The mean age was 51.9 ± 10.7 years (range, 25–74 years). The mean follow-up period for disease-free survival was 54.2 ± 43.2 months (range, 5–154 months). The total number of recurrences of breast cancer during this period was 19 (40.4%). The number of subjects with local recurrence, regional recurrence, loco-regional recurrence, and systemic recurrence were 3, 8, 10, and 15, respectively.

### Comparison of Tissue Microbiota across Normal, Tumor, and Lymph Node Samples

**Fig. 1:**
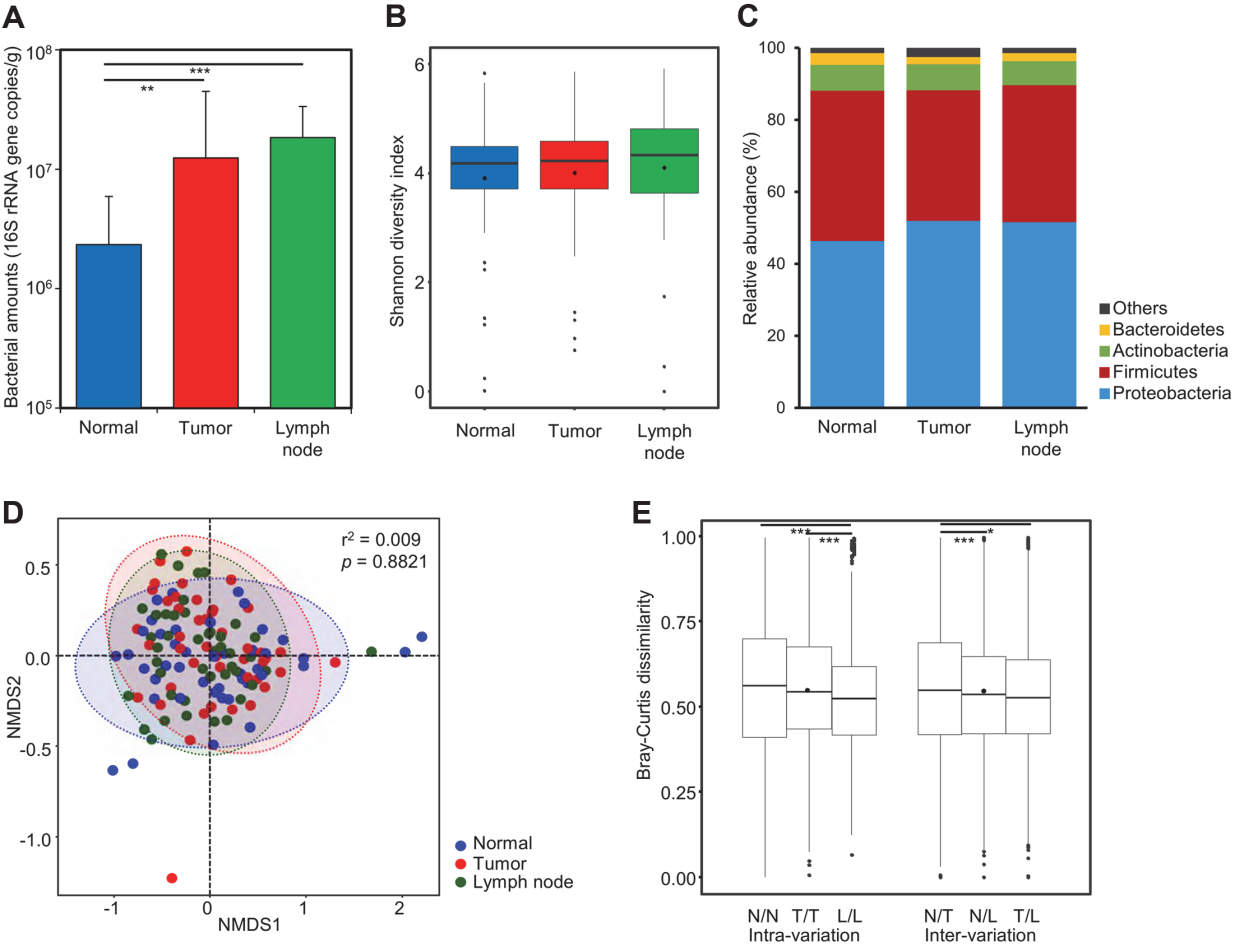
Comparison of microbiota across normal, tumor, and lymph node tissues. (**A**) The relative bacterial amounts were compared across tissue types. The bacterial amounts were determined by quantitative real-time PCR based on 16S rRNA gene. (**B**) The Shannon diversity indices in microbiota were compared across tissue types. (**C**) Comparison of phylum composition across tissue types. (**D**) Microbiota across tissue types was compared using NMDS plot. (**E**) Intravariation of microbiota within the same tissue and inter-variation of microbiota across tissue types were compared based on Bray-Curtis dissimilarity. *p < 0.05, **p < 0.01, ***p < 0.001.

The relative bacterial amounts in obtained samples were significantly different across tissue types (Fig. 1A; *p* < 0.01). The highest bacterial amount was detected in lymph node tissue samples (average, 1.13 × 10^7^ cells/g), and the lowest was detected in normal tissue (6.84 × 10^6^ cells/g).

A total of 5,020,282 reads (1,575,709 reads for normal tissues, 1,665,271 reads for tumor tissues, and 1,779,302 reads for lymph node tissues) were analyzed after trimming processes. Bacterial sequences were detected in negative controls as previous report (Fig. S1) [41]. However, the microbiota in negative controls were distinguished to those in tissue samples (*p* < 0.001). We analyzed the microbiota in tissue samples after removing sequence reads in negative controls. Diversity indices were compared across samples after the normalization of read numbers (Table S1). Shannon diversity indices were similar across tissue types (Fig. 1B; *p* > 0.05). Although the mean value of each phylum was different across tissue types, the difference was not statistically significant (Fig. 1C; *p* > 0.05). The NMDS plot also showed the microbiota of breast tissue samples to not be simply distinguishable based on tissue types (Fig. 1D; *p* > 0.05). The intra-variation of microbiota in each tissue and inter-variation of microbiota across tissue types were compared based on Bray-Curtis dissimilarity (Fig. 1E). The intra-variation of microbiota within lymph node tissue was lower than that in normal and tumor tissues (*p* < 0.001). The inter-variation of microbiota between normal and tumor tissues was higher than that between lymph node and normal or tumor tissues (*p* < 0.05). However, the tissue microbiota could not be clearly distinguished by tissue types. In addition, we analyzed the difference in microbiota based on clinical characteristics, such as stage, HR, and ER, among others, and did not find significant differences across tissue types.

### Differences in Microbiota within the Same Tissue Type

**Fig. 2:**
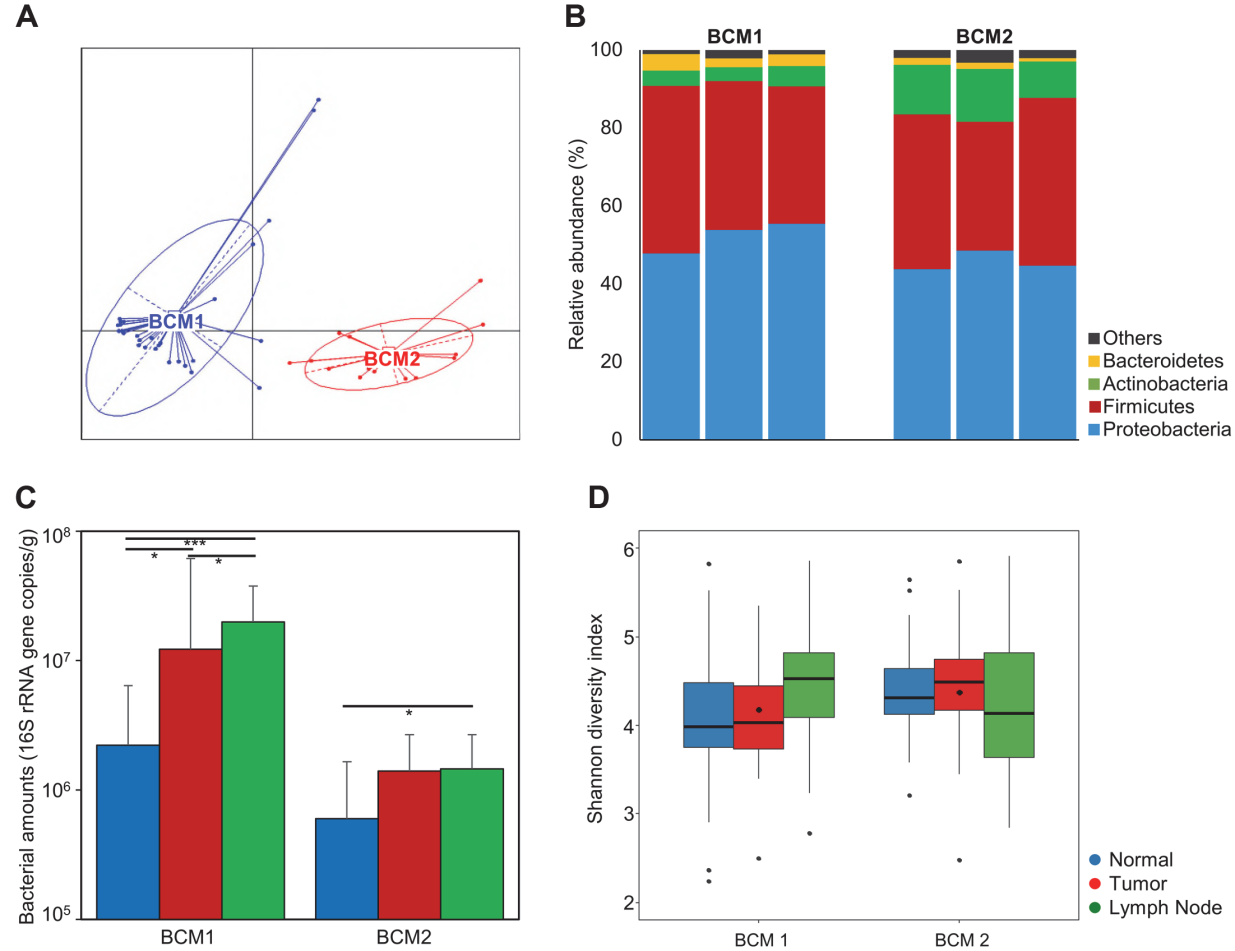
Comparison of microbiota between BCM1 and BCM2. (**A**) Cluster analysis of microbiota in tumor tissues by PAM clustering using Jensen-Shannon divergence (JSD) based on the relative abundance of genus. (**B**) Comparison of phylum composition across tissue types between BCM1 and BCM2. (**C**) Comparison of bacterial amounts across tissue types between BCM1 and BCM2. (**D**) Comparison of Shannon diversity indices across tissue types between BCM1 and BCM2. *p < 0.05, ***p < 0.001.

The microbiota in tissue samples was not distinguishable based on tissue types; thus, we analyzed the differences in tissue microbiota within the same tissue-type by enterotype clustering. Since the patients had cancer, clustering was performed by the PAM clustering method using Jensen-Shannon divergence based on the relative abundance of genera in tumor tissue. The optimal number of clusters was determined by the CH index score. Microbiota from tumor tissues were separated into two different clusters (Fig. 2A). Breast cancer microbiota cluster 1 (BCM1) contained samples from 30 subjects, and breast cancer microbiota cluster 2 (BCM2) contained samples from 17 subjects. Compositions at the phylum level were compared across the tissue types in both BCM1 and BCM2 (Fig. 2B). This was not significantly different across the tissue types within each cluster (*p* > 0.05). However, a significant difference was detected between BCM1 and BCM2. The relative abundances of *Actinobacteria* were significantly higher in all tissues (12.7% in normal, 13.6% in tumor, and 9.4% in lymph node) of BCM2 than in BCM1 (4.0%, 3.6%, and 5.2%, respectively; *p* < 0.01). In contrast, the relative abundances of *Proteobacteria* and *Bacteroidetes* were higher in lymph node tissue (55.4% and 3.0%) of BCM1 than those of in BCM2 (44.6% and 0.9%; *p* < 0.05).

The differences in microbiota were also detailed at the genus level. The frequently detected genera (> 1% of total microbiota in each sample) were compared across tissue types (Fig. S2). Genus compositions were different between BCM1 and BCM2 clusters. The proportions of *Brochothrix* and *Moraxella* were higher in the tissue of BMC1 than in that of BCM2 cluster (*p* < 0.001), whereas *Enterococcus*, *Cutibacterium*, and *Escherichia* were more abundant in tissues of BCM2 than in those of BCM1 (*p* < 0.05). This result indicated that the microbiota in tissues from patients with breast cancer was not significantly different across tissue types and rather differed by cluster type.

The relative bacterial amounts were also compared across tissue types, in each cluster, and between clusters (Fig. 2C). The bacterial amounts were higher in all tissues of BCM1 than in those of BCM2 (*p* < 0.01). The bacterial amounts in lymph node (average 1.38 × 10^7^ cells/g) and tumor tissues (1.25 × 10^7^ cells/g) were higher than those in normal tissues (8.08 × 10^6^ cells/g) in the BCM1 cluster (*p* < 0.01). Similar results (6.98 × 10^6^ cells/g for lymph node, 6.88 × 10^6^ cells/g for tumor, and 4.67 × 10^6^ cells/g for normal tissues) were detected in the BCM2 cluster (*p* < 0.05 between normal and lymph node tissues). Bacterial diversity was compared across tissue types in each cluster (Fig. 2D). Although diversity was the highest in lymph node tissues, as seen from bacterial amounts in the BCM1 cluster, the differences were not significant (*p* > 0.05). Diversity was lowest in lymph node tissue of the BCM2 cluster, although differences in diversity indices across tissue types were not significant (*p* > 0.05). The diversity indices in the same tissue type between BCM1 and BCM2 were also not significant.

### Clinicopathologic Characteristics and Survival Analyses according to BCM1 and BCM2

Clinicopathological characteristics of subjects in BCM1 and BCM2 are summarized in Table 1. Subjects in the BCM2 cluster showed significantly higher ratios of HG 3 (*p* = 0.020) and NG 3 (*p* = 0.033) compared to those in BCM1 cluster. No significant differences were observed in the other clinicopathologic factors, including age, TNM staging, HR, HER2, lymphovascular invasion, body mass index, and treatment modalities.

**Fig. 3:**
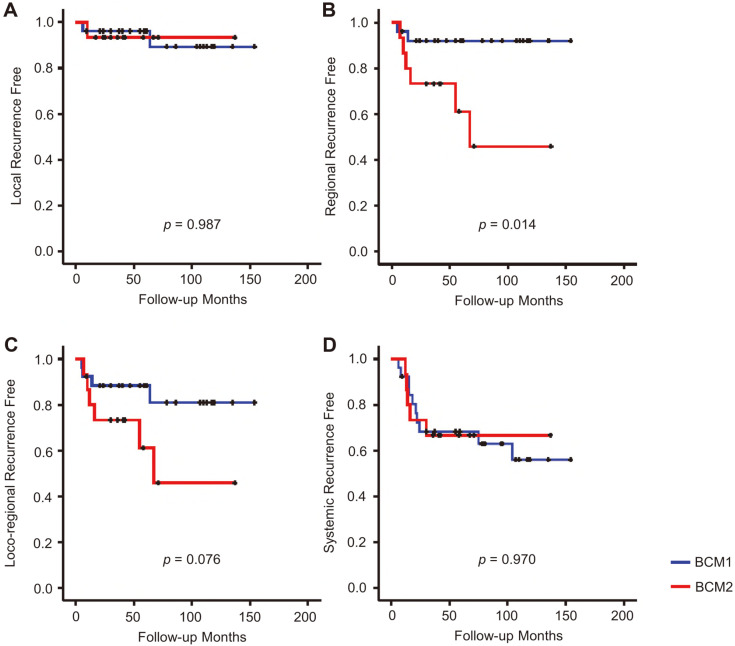
Recurrence curves were compared between BCM1 and BCM2. (**A**) Local recurrence, (**B**) regional recurrence, (**C**) loco-regional recurrence, and (**D**) systemic recurrence were compared.

Survival analyses showed that the BCM2 group had worse regional recurrence-free survival than the BCM1 group (*p* = 0.014; Fig. 3). No significant survival difference was observed regarding local recurrence, loco-regional recurrence, and systemic recurrence (Fig. 3). There were no significant survival differences regarding overall survival and disease-free survival between the two groups (Fig. S3).

**Fig. 4:**
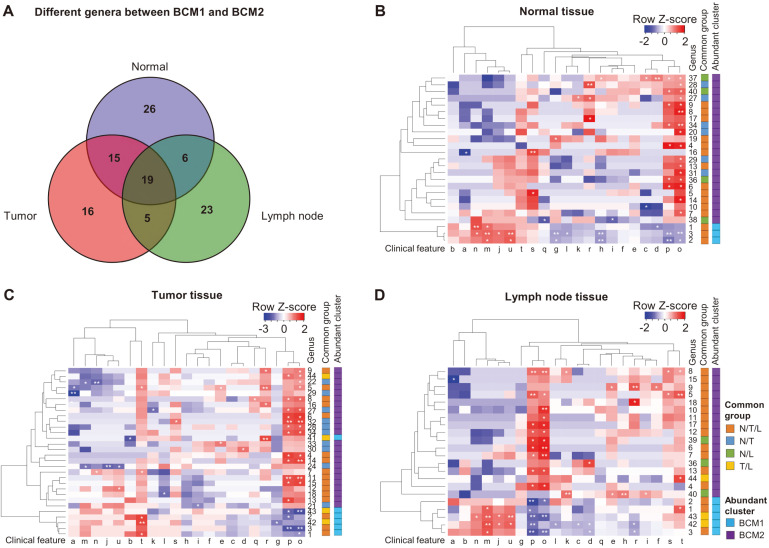
Different genera across tissue types, between BCM1 and BCM2, and their correlation with clinical features. (**A**) The number of commonly different genera across tissue types between BCM1 and BCM2. Correlations of clinical features with commonly different genera (**B**) in normal tissue, (**C**) tumor tissue, and (**D**) lymph node tissue were compared in heatmap. Genus number, 1: *Myroides*; 2: *Moraxella*; 3: *Brochothrix*; 4: *Enterococcus*; 5, *Cutibacterium*; 6: *Lactobacillus*; 7: *Escherichia*; 8: *Micrococcus*; 9: *Corynebacterium*; 10: *Uncultured_Caulobacteraceae*_JPOM; 11: *Streptococcus*; 12: *Stenotrophomonas*; 13: *Achromobacter*; 14: *Alishewanella*; 15: *Azotobacter*; 16: *Ralstonia*; 17: *Haemophilus*; 18: *Thauera*; 19: *Labilithrix*; 20: *Altererythrobacter*; 21: *Uncultured_Sandaracinaceae*_JF344203; 22: *Comamonas*; 23: *Chryseomicrobium*; 24: *Pseudoxanthomonas*; 25: *Microbacterium*; 26: *Brevundimonas*; 27: *Gemmobacter*; 28: *Prevotella*; 29: *Curvibacter*; 30: *Afipia*; 31: *Methylobacterium*; 32: *Aquabacterium*; 33: *Uncultured_Neisseriaceae*_FM873692_g; 34: *Deinococcus*; 35: *Burkholderia*; 36: *Akkermansia*; 37: *Bacillus*; 38: *Coprococcus*; 39: *Veillonella*; 40: *Bradyrhizobium*; 41: *Rhizobium*; 42: *Carnobacterium*; 43: *Hafnia*; 44: *Porphyrobacter*; 45: *Sporobacter*. Clinical features, a: Age; b: BMI; c: T stage; d: T stage 012/34; e: N stage; f: N stage 01/2 e; i: All stage 012/34; j: HR; k: HER2; l: Subtype; m: ER; n: PgR; o: HG; p: NG; q: LI; r: VI; s: RT; t: CT; u: ET. *p < 0.05, **p < 0.01, ***p < 0.001.

Differences in Microbiota between BCM1 and BCM2 Clusters and Their Correlation with Clinical Features Tissue microbiota was not clearly distinguishable based on tissue types in each cluster; thus, we focused on the differences in microbiota between the clusters. The significantly different genera between BCM1 and BCM2 were determined using the LEfSe program, and the linear discriminant analysis (LDA) score was over 2.4. Sixty-six genera in normal tissues, 55 in tumor tissues, and 53 in lymph node tissues were different between BCM1 and BCM2 (Fig. 4A). Forty-five genera were commonly different between the clusters in at least two tissue types, and 19 genera were commonly different in all tissues. Among the 19 commonly different genera in all tissues, the relative abundances of three genera (*Brochothrix*, *Moraxella*, and *Myroides*) were higher in BCM1 than in BCM2, and those of the other 16 genera, including *Enterococcus* and *Cutibacterium*, were higher in BCM2 (Fig. S4). The correlation between commonly different genera and clinical features in each tissue was analyzed by Spearman’s correlation (Figs. 4B-4D). Similar correlations between clinical features and abundant genera in each cluster were detected across all tissue types. In particular, HG and NG were positively correlated with the abundant genera of BCM2, whereas they were negatively correlated with the abundant genera of BCM1 in all tissue types (*p* < 0.05). This result was consistent with clinicopathologic characteristics between BCM1 and BCM2 (Table 1). *Myroides*, *Moraxella*, and *Brochothrix*, which were more abundant in BCM1 than in BCM2, were negatively correlated with HG and NG, whereas *Enterococcus*, *Lactobacillus*, *Micrococcus*, *Corynebacterium*, *Escherichia*, and *Achromobacter*, which were more abundant in BCM2 than in BCM1, were positively correlated with HG and NG (*p* < 0.05). Although the significance of correlations was different based on tissue types, the inverse correlation with HG and NG, between BCM1 and BCM2, showed that the two factors are important to determine the microbiota type. The abundant genera in BCM1 were positively correlated with HR, ER, PgR, and endocrine therapy (ET) in normal and lymph node tissues. In tumor tissues, the abundant genera in BCM1 were positively correlated with chemotherapy. *Comamonas* and *Pseudoxanthomonas*, which were abundant in BCM2, were negatively correlated with HR, ER, PgR, and ET in tumor tissues.

### Comparison of The Potential Functions of Tissue Microbiota between BCM1 and BCM2

**Fig. 5:**
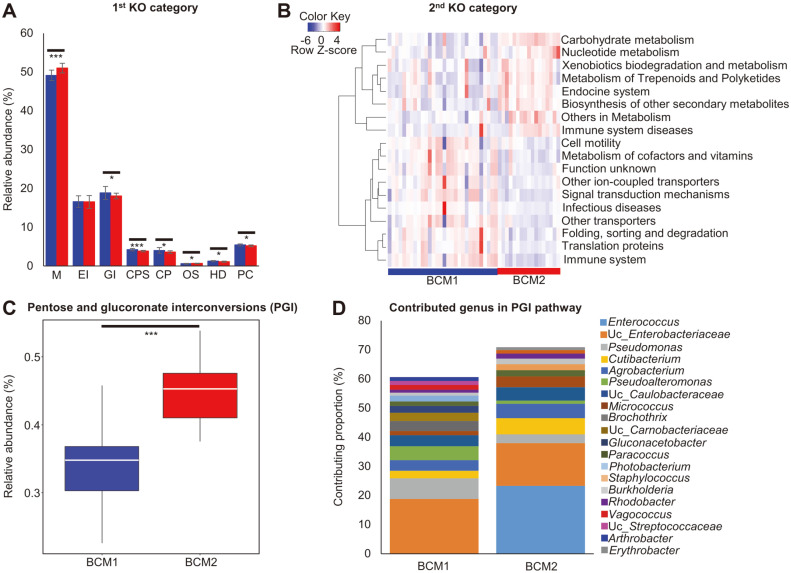
Comparison of KEGG pathways between BCM1 and BCM2. (**A**) 1^st^ KO category and (**B**) 2^nd^ KO category. The significantly different categories were selected by Benjamini-Hochberg FDR adjusted *q* < 0.05. (**C**) Relative abundance of pentose and glucuronate interconversions was compared between BCM1 and BCM2 in boxplot. (**D**) The contributing genera in PGIs pathway were compared between BCM1 and BCM2. *q < 0.05, ***q < 0.001.

**Fig. 6:**
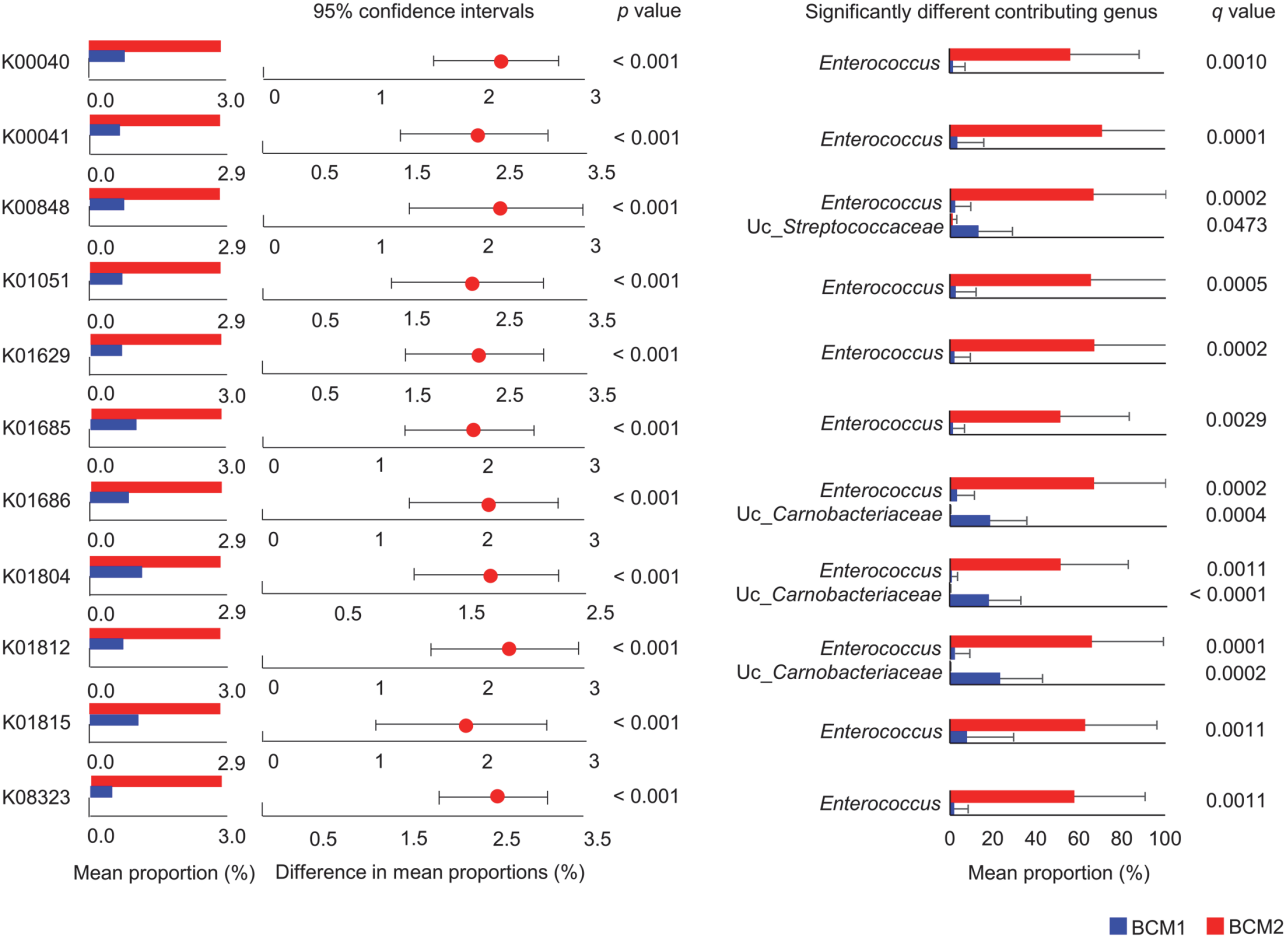
The predicted KEGG orthologs (KO) of microbiota. Significantly different KO were selected by the Kruskal- Wallis H-test, and a post-hoc test was performed using the Tukey-Kramer method. Significantly different contributing genera were determined by Welch’s *t*-test with Benjamini-Hochberg FDR correction.

The potential functions of microbiota were predicted by PICRUSt2 and compared between BCM1 and BCM2. The predicted functions were analyzed according to KEGG pathway hierarchies. Seven categories within the 1^st^ KEGG Orthology (KO) classification were significantly different between the clusters (Fig. 5A; *q* < 0.05). Sixty-three categories were detected in the 2^nd^ KO category, and 18 categories were significantly different between the clusters (Fig. 5B; *q* < 0.05). The differences in the 18 categories are shown by heatmap analysis. The relative abundances of eight categories, including carbohydrate metabolism and nucleotide metabolism, were higher in BCM2 than in BCM1, and those of the other 10 categories were higher in BCM1. Next, we detailed the potential functions of microbiota in the 3^rd^ KO category and a total of 328 categories were detected. Eleven pathways were selected as significantly different between the clusters (*q* < 0.001 and effect size > 0.4; Table S2). Pentose and glucuronate interconversions (PGIs), other glycan degradation, polycyclic aromatic hydrocarbon degradation, glycosphingolipid biosynthesis, and galactose metabolism were significantly higher in BCM2 than in BCM1. The effect size of PGIs was the highest among them, and relative abundance was significantly higher in BCM2 than in BCM1 (Fig. 5C). The contributing genera for the PGIs pathway were different, and *Enterococcus* was the major contributor only in BCM2. KO identifiers (gene families) were then compared between BCM1 and BCM2 using post-hoc analysis, and 11 functional orthologs were detected to be significantly higher in BCM2 than in BCM1 (Fig. 6). Differences in these orthologs could be caused by the contribution of *Enterococcus* in BCM2. Seven functional orthologs were selected based on effect size (> 0.4) from 11 orthologs in the post-hoc analysis, and a significantly different pathway map of PGIs between the two clusters was predicted (Fig. S5). While D-galacturonate could be degraded more, D-glucuronate could be generated more in BCM2, than in BCM1, by the breast tissue microbiota.

## Discussion

The present study analyzed the microbiota of normal, tumor, and lymph node tissues from the same patient with breast cancer in Korea and compared them in terms of clinicopathological features and potential functions. The microbiota was not simply distinguishable based on tissue types and could be separated into two clusters, even within the same tumor tissue. The associations between microbiota and clinicopathologic factors, regional recurrence, and potential functions were significantly different between the two microbiota clusters.

*Proteobacteria* and *Firmicutes* were the dominant phyla in tumor, adjacent normal, and lymph node tissues, and there were no significant differences in microbiota and Shannon diversity index across the tissues (Fig. 1). The intra-variation of microbiota within the same tissue type was similar to the inter-variation among different tissue types. This could be the reason for the lack of a significant difference in microbiota across the tissues. The dominance of *Proteobacteria* and *Firmicutes* in breast tissue microbiota had been reported in previous studies, regardless of geographical or racial differences [12, 17, 19, 25]; the fatty acid-rich environment of the breast and microbial adaptation to this environment were suggested as the underlying reasons [17]. Urabaniak *et al*. reported that the microbiota in tumor tissue does not differ from that in adjacent normal tissue, although it was remarkably different from the breast tissue of healthy subjects [17, 19]. Results from the present study were consistent with these previous studies. Since normal breast tissues from healthy subjects were not included in this study, we could not find a difference in breast microbiota between healthy subjects and patients with breast cancer. A previous study reported bacterial amounts in tumor tissue to be lower than those in paired normal tissue, and the amounts were reduced based on breast cancer stage in tumor tissue [12]. However, the bacterial amounts in normal tissue were found to be lower than those in tumor and lymph node tissues in the present study. Such discrepancies between studies could be due to the differences in tissue sample preparation, sample subgroups (stage or grade), experimental factors (such as the primers used), analysis pipeline, geographical location, and race. Complex environmental factors can influence the human microbiome; thus, geographical and racial differences constitute an important factor in determining microbiome results [17, 19, 25]. Breast tissue microbiome studies should consider these factors and extend this information, since the risk of breast cancer varies across races [1, 42, 43].

Although the microbiota was not different across tissue types, we found it to be significantly distinguishable into two different clusters (BCM1 and BCM2) within the same tumor tissue (Fig. 2). Significantly different phyla (*Actinobacteria*, *Proteobacteria*, and *Bacteroidetes*) and genera (*Brochothrix*, *Moraxella*, *Enterococcus*, *Cutibacterium*, and *Escherichia*) were detected between BCM1 and BCM2 (*p* < 0.05). However, the difference in microbiota composition across tissue types was not significant in each cluster (*p* > 0.05). Next, we focused on the differences and potential influences of microbiota between BCM1 and BCM2. While comparing clinical features based on microbiota clusters, we found the regional recurrence to be significantly increased in BCM2 compared to that in the BCM1 group (Fig. 3). This has remarkable implications, since the ratio of regional recurrence was significantly different between subject groups determined by microbiota cluster. This result indicated that the microbiota of BCM2 was associated with a higher risk of regional recurrence. The commonly different genera across tissue types, between BCM1 and BCM2, were determined by LEfSe analysis, and similar correlations between different genera and clinical features were detected in all tissue types (Figs. 4 and S4). HG and NG were significantly correlated with commonly different genera, between BCM1 and BCM2, and their correlations were opposite between the two clusters. These results indicated that the breast tissue microbiota could interact with host factors and that different microbiota could influence the progression of breast cancer differently.

The potential influence of microbiota on breast cancer, between BCM1 and BCM2, was compared by functional prediction (Fig. 5). The functions of microbiota were predicted to be different between BCM1 and BCM2 based on KEGG categories. Significantly different pathways at the 3^rd^ KEGG category level, between the clusters, were determined by FDR- adjusted *q* value and effect size, and the PGIs pathway was significantly more represented in BCM2 than in BCM1 (Fig. 5 and Table S2). The differences in this pathway between the clusters were caused by different genera. The PGIs pathway is involved in interconversions of the monosaccharide pentose and glucuronate and is closely related to glycolysis and gluconeogenesis. Dysregulation of PGIs in various cancers, including breast cancer, has been detected and reported in previous studies [44-46]. Gene families in this pathway were different between BCM1 and BCM2, and *Enterococcus* in BCM2 contributed significantly to this difference (Fig. 6). These results indicated that the different microbiota are related to the dysregulation of PGIs in breast cancer and could be associated with the risk of regional recurrence in patients. *Enterococcus* is a facultative anaerobe that can survive under a wide range of environmental conditions [47]. *Enterococcus* sp. can express toxins, such as lysin and hemolysin, and produce gelatinase and serine protease, which can degrade host tissues and promote microbial invasion [48]. However, the role of *Enterococcus* is still unclear and controversial. Some studies have reported that species of *Enterococcus* show anti-tumor effects, as in probiotics [49, 50], whereas other studies have reported these bacteria to be harmful in cancer due to their ability to generate reactive oxygen species (ROS) and extracellular superoxide, which can cause genomic instability and damage host cell DNA [51, 52]. The role of *Enterococcus* should be investigated in further detail and under different experimental conditions. A potential differential pathway map by breast tissue microbiota was constructed based on significantly different orthologs between BCM1 and BCM2 (Fig. S5). These orthologs were predicted to be higher in BCM2 than in BCM1. D-galacturonate could be degraded more in BCM2, whereas D-glucuronate could either be generated more or degraded in BCM2. D-glucuronate is a precursor of UDP-D-glucuronate in this pathway. UDP-D-glucuronate was found to be higher in tumor tissues than in normal tissues of patients with breast cancer, and this metabolite could promote tumorigenesis [53, 54]. Therefore, the dysregulation of D-glucuronate in BCM2 by microbiota could be related to the recurrence of breast cancer. The relative abundances of other glycan degradation, glycosphingolipid biosynthesis, and galactose metabolism were predicted to be higher in BCM2 than in BCM1 (Table S2). These pathways are related to the biosynthesis of glycoproteins and glycolipids, which are involved in cancer progression, through the dysregulation of cell cycle, promotion of tumor dissemination and angiogenesis, and facilitation of immune evasion [55-57]. Glyco-conjugated proteins act as receptors for bacteria and function in cell–cell and cell–matrix interactions, which are associated with cancer–cell invasion to the surrounding tissue or extravasation to form metastatic lesions [58]. The higher abundances of these pathways in BCM2, compared to those in BCM1, could be related to the risk of cancer progression through host cell dysregulation by microbiota. Although these predicted results should be validated in further studies, possible roles of breast tissue microbiota were discussed in the present study.

The limitation of this study was the relatively small number of subjects and lack of comparison of breast tissue microbiota between patients and healthy subjects; however, this study analyzed the microbiota of tumor, adjacent normal, and lymph node tissues of female Korean patients. In addition, we removed potential background signals from tissue samples by sequencing negative control samples. This is critical to analyze microbiota results in low-biomass samples using amplicon-based sequencing [41]. Further studies, including metabolomics, whole metagenomics, and host interactions, would be necessary to validate the results of this study. Furthermore, analysis pipeline based on DADA2 can improve OTU clustering results [59], thus we will apply updated pipeline to further studies. Although generalization of the results in this study could be difficult due to these limitations, this study could extend our understanding about the microbiota in breast tissues and their potential roles in patients with breast cancer.

In summary, the tissue microbiota were not significantly different across tissue types (tumor, adjacent normal, and lymph node); however, they were significantly clustered into two types (BCM1 and BCM2) even within the same tissue. The correlation between clinical features and microbiota was different between BCM1 and BCM2, and regional recurrence was found to be higher in subjects with the BCM2 type. The results of functional prediction showed that the dysregulation of PGIs was related to the incidence of regional recurrence in BCM2 by *Enterococcus* in tissue microbiota. These results indicated that the microbiota in breast tissue play a role in the development and progression of breast cancer. Results from this study might help to expand our understanding of breast tissue microbiota in patients with breast cancer and to develop novel biomarkers for its recurrence and treatment.

## Supplemental Materials

Supplementary data for this paper are available on-line only at http://jmb.or.kr.

## Figures and Tables

**Table 1:** Clinicopathologic characteristics in BCM1 and BCM2.

Characteristic	All		BCM1		BCM2		χ^2^ test

	No.	%	No.	%	No.	%	*p* ^a)^
Total	47	100.0	30	63.8	17	36.2	
Mean age (years)	51.9±10.7		54.1±11.0		48.1±9.3		0.065
Age (years)							0.214
≤ 50	22	46.8	12	40.0	10	58.8	
> 50	25	53.2	18	60.0	7	41.2	
Tumor size (cm)							0.447
≤ 2	1	2.1	1	3.3	0	0.0	
> 2	46	97.9	29	96.7	17	100.0	
Nodal positivity							
Negative	0	0.0	0	0.0	0	0.0	
Positive	47	100.0	30	100.0	17	100.0	
Distant metastasis							0.877
Negative	41	87.2	26	86.7	15	88.2	
Positive	6	12.8	4	13.3	2	11.8	
Stage							0.962
Stage II	9	19.1	6	20.0	3	17.6	
Stage III	32	68.1	20	66.7	12	70.6	
Stage IV	6	12.8	4	13.3	2	11.8	
Hormone receptor							0.468
Negative	17	36.2	12	40.0	5	29.4	
Positive	30	63.8	18	60.0	12	70.6	
Estrogen receptor							0.937
Negative	19	40.4	12	40.0	7	41.2	
Positive	28	59.6	18	60.0	10	58.8	
Progesterone receptor							0.979
Negative	22	46.8	14	46.7	8	47.1	
Positive	25	53.2	16	53.3	9	52.9	
HER2							0.534
Negative	33	70.2	22	73.3	11	64.7	
Positive	14	29.8	8	26.7	6	35.3	
Histologic grade							0.020
1,2	12	25.5	11	36.7	1	5.9	
3	35	74.5	19	63.3	16	94.1	
Nuclear grade							0.033
1,2	11	23.4	10	33.3	1	5.9	
3	36	76.6	20	66.7	16	94.1	
Lymphovascular invasion							0.470
Negative	8	17.0	6	20.0	2	11.8	
Positive	39	83.0	24	80.0	15	88.2	
Body mass index (kg/m^2^)							0.589
≤ 25	28	59.6	17	56.7	11	64.7	
> 25	19	40.4	13	43.3	6	35.3	
Operation							
Lumpectomy	0	0.0	0	0.0	0	0.0	
Mastectomy	47	100.0	30	100.0	17	100.0	
Radiation therapy							0.353
Yes	15	31.9	11	36.7	4	23.5	
No	32	68.1	19	63.3	13	76.5	
Chemotherapy							0.627
Yes	4	8.5	3	10.0	1	5.9	
No	43	91.5	27	90.0	16	94.1	
Herceptin therapy							0.305
No	37	78.7	25	83.3	12	70.6	
Yes	10	21.3	5	16.7	5	29.4	
Endocrine therapy							0.345
Yes	18	38.3	13	43.3	5	29.4	
No	29	61.7	17	56.7	12	70.6	

Abbreviations: BCM1, breast cancer microbiota cluster 1; BCM2, breast cancer microbiota cluster 2; HER2, human epidermal growth factor receptor 2

^a^p value for mean age was calculated by *t*-test and all the other *p* values were calculated by *χ*^2^ test.
