# Supplementary material for: Microbiota of Breast Tissue and Its Potential Association with Regional Recurrence of Breast Cancer in Korean Women
Source: J Microbiol Biotechnol. 2021 Sep 25;31(12):1643–55. doi: 10.4014/jmb.2106.06039 (PMC9705848; doi:10.4014/jmb.2106.06039)
Supplement: Supplementary file 1 [file jmb-31-12-1643-supple.pdf]

J. Microbiol. Biotechnol.
https://doi.org/10.4014/jmb.2106.06039

Supplementary Materials

Microbiota of Breast Tissue and Its Potential Association with Regional Recurrence of Breast Cancer in Korean Women

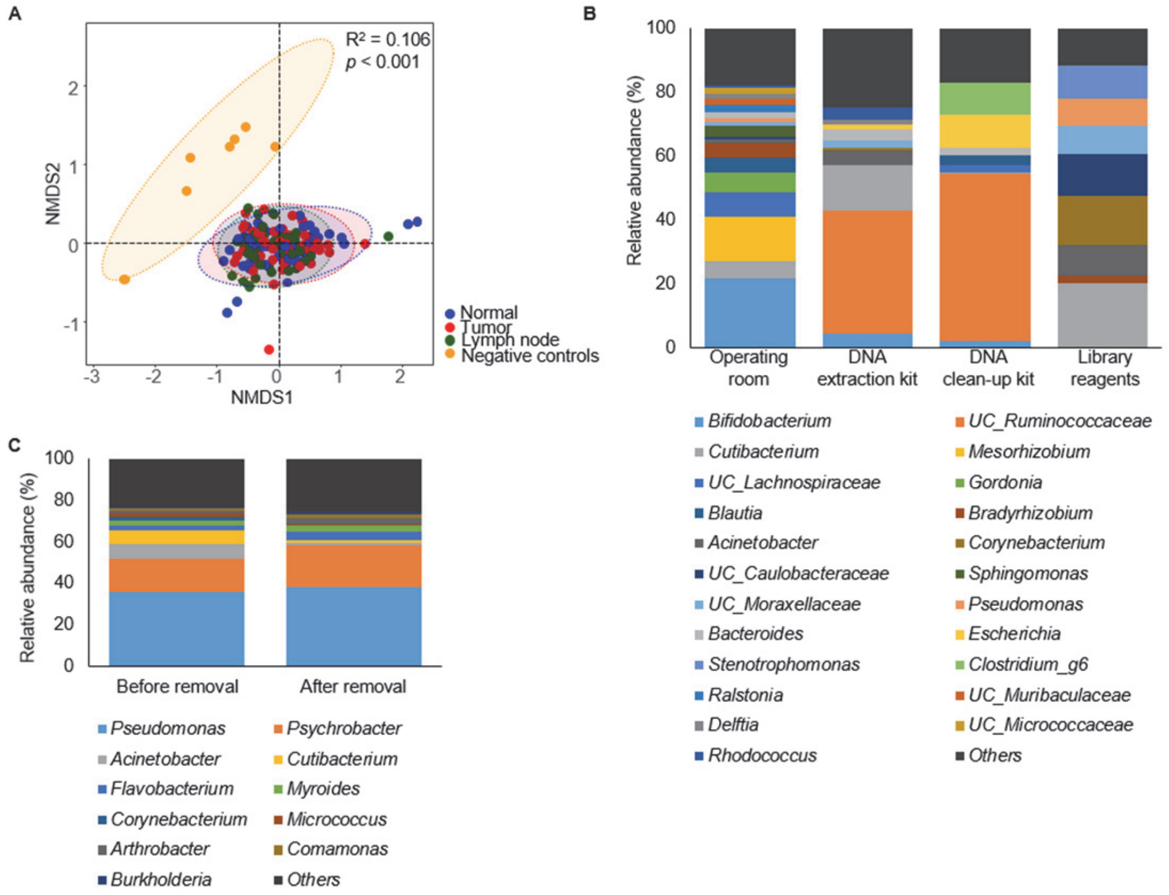

Fig. S1. Microbiota detected in negative control samples. (A) Differences of microbiota between negative controls and tissue samples. (B) The composition of genus detected in negative controls. (C) Comparison of genus composition between before and after removal sequences reads in negative controls from tissue samples.

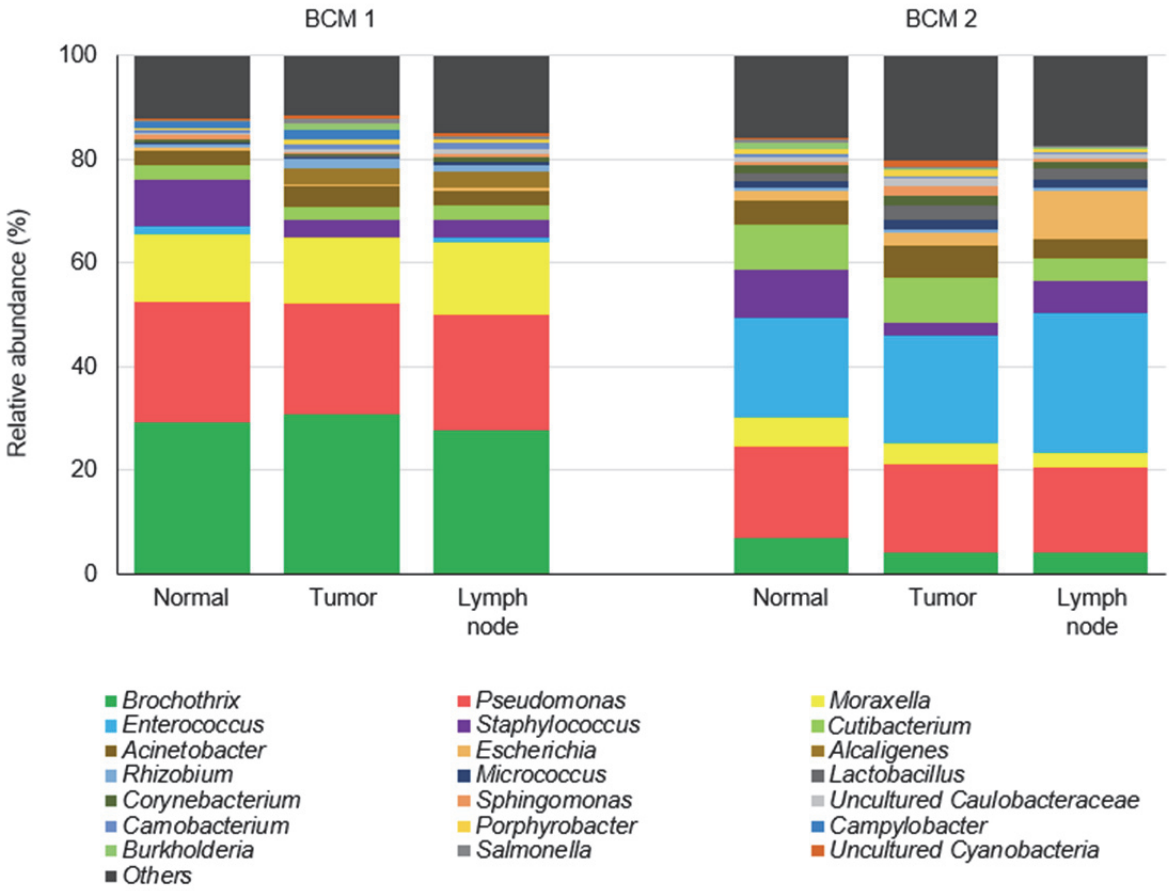

Fig. S2. The comparison of genus in three different tissue types between BCM1 and BCM2.

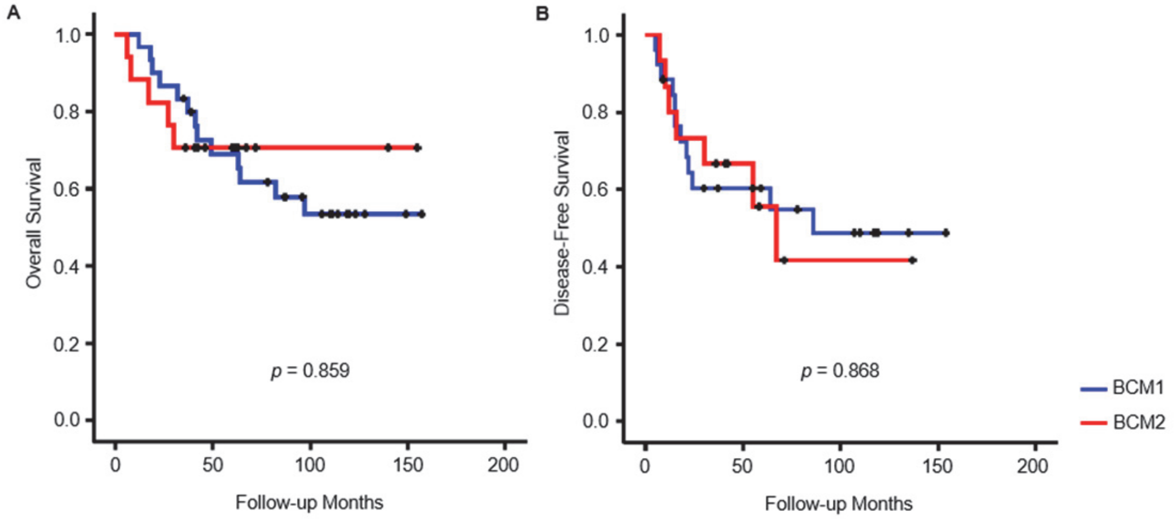

Fig. S3. Overall survival curves and disease-free survival curves in BCM1 and BCM2.

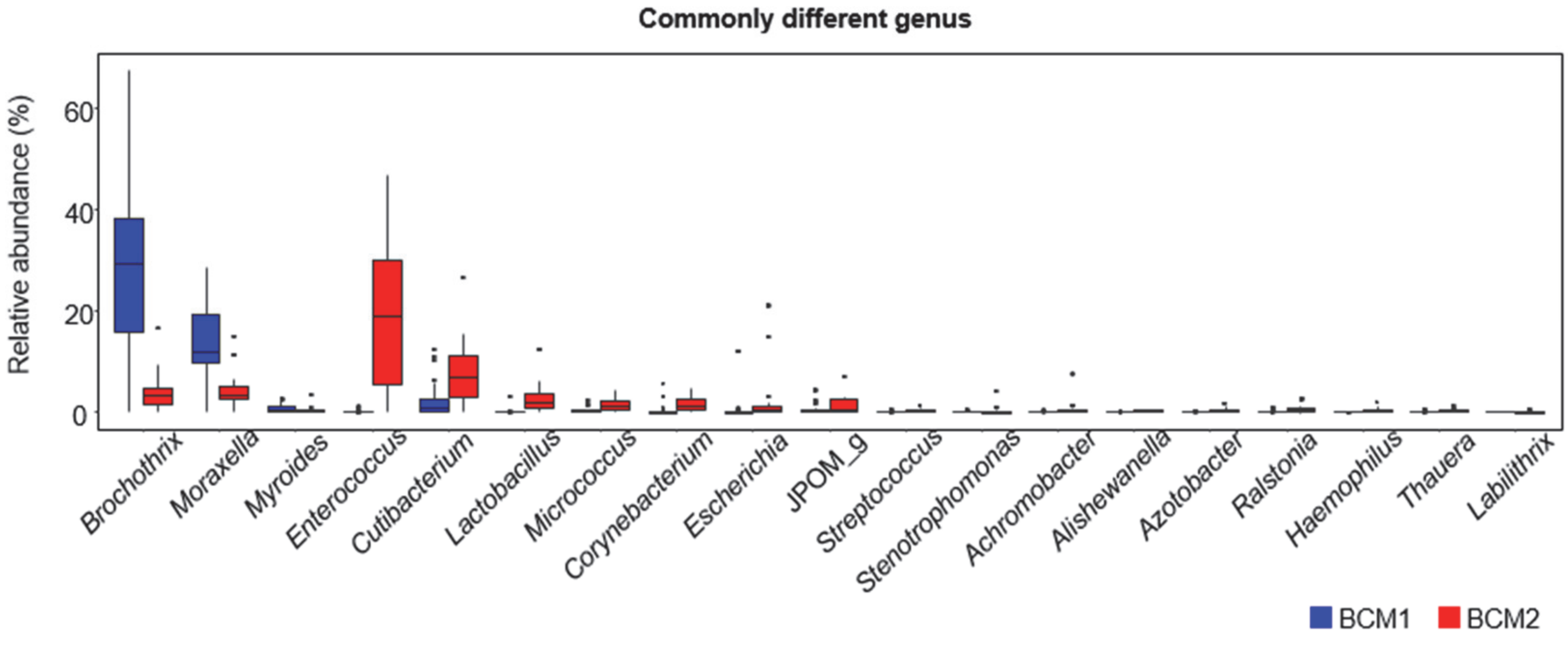

Fig. S4. Relative abundances of commonly different genera in all tissues between BCM1 and BCM2.

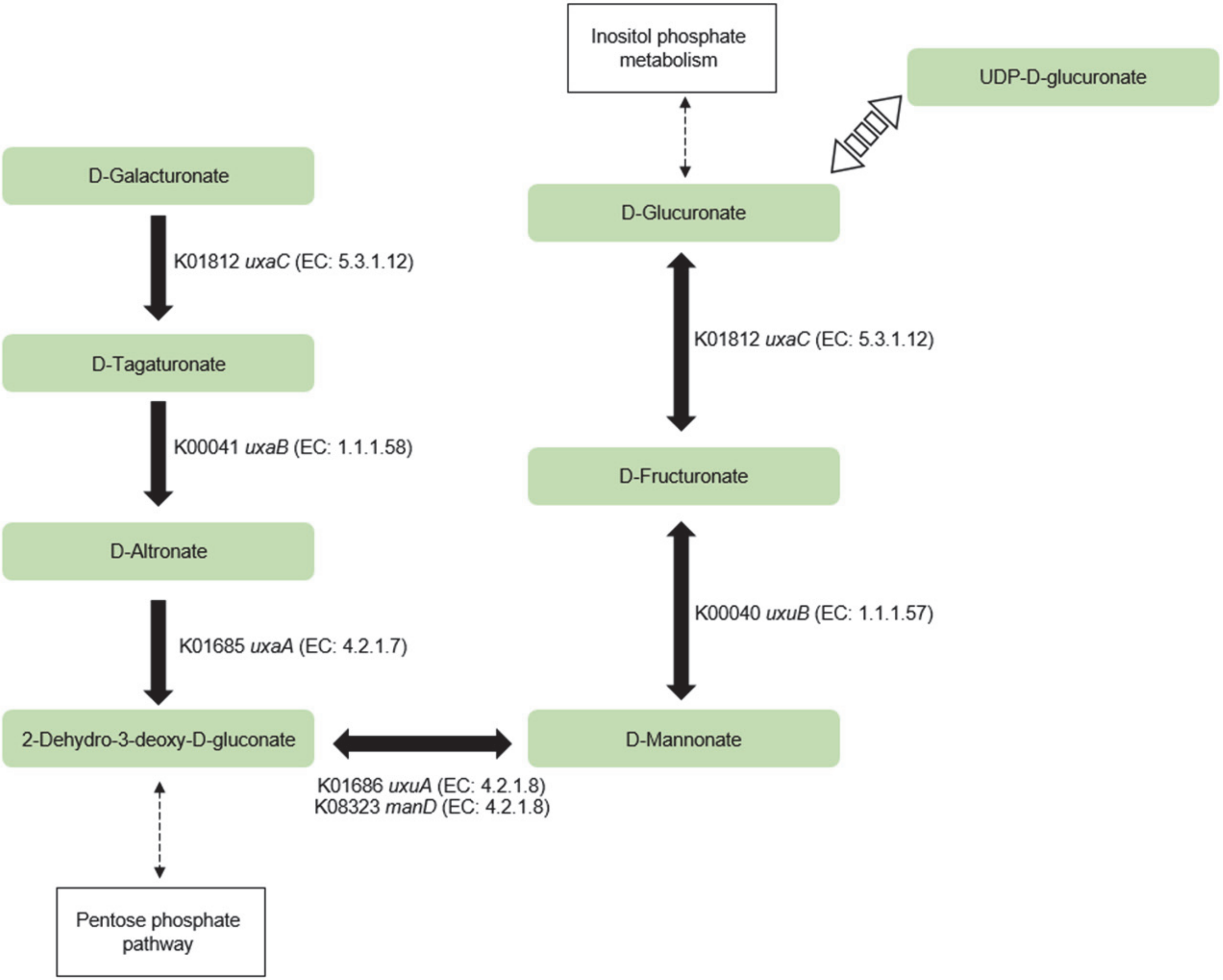

Fig. S5. Potential metabolic pathway significantly different between BCM1 and BCM2.

Table S1. Summary of diversity indices after normalizing read number.

Subject no.	Tissue type	Analyzed reads	Normalized reads	Observed OTUs	Estimated OTUs (Chao1)	Shannon diversity index	Good's coverage
1	Normal tissue	8,409	5,100	365	739.62	3.74	0.97
2		15,422	5,100	350	1,006.38	3.64	0.97
3		13,523	5,100	394	1,017.56	4.04	0.96
4		6,137	5,100	107	174.56	1.34	0.99
5		32,704	5,100	385	740.32	4.40	0.96
6		27,658	5,100	295	521.48	4.44	0.98
7		28,795	5,100	41	71.00	0.24	0.99
8		27,132	5,100	523	951.84	4.97	0.95
9		12,926	5,100	1,379	4,952.39	5.52	0.81
10		23,662	5,100	1,556	5,241.60	5.83	0.78
11		41,811	5,100	761	1,571.27	4.65	0.92
12		24,421	5,100	30	43.75	1.22	0.99
13		18,331	5,100	673	1,703.32	4.49	0.92
14		11,792	5,100	571	1,274.25	4.49	0.94
15		18,880	5,100	479	976.02	3.86	0.96
16		50,677	5,100	4	4.50	0.01	0.99
17		30,151	5,100	531	1,247.33	3.77	0.94
18		10,811	5,100	468	983.31	3.93	0.95
19		15,579	5,100	925	2,954.67	4.67	0.88
20		23,481	5,100	566	989.90	4.20	0.95
21		28,183	5,100	570	1,459.84	4.07	0.94
22		6,655	5,100	181	341.20	2.36	0.98
23		7,430	5,100	485	1,177.52	3.68	0.95
24		15,518	5,100	472	1,064.93	4.19	0.96
25		13,930	5,100	216	381.45	2.23	0.98
26		13,896	5,100	274	505.62	3.58	0.98
27		7,359	5,100	264	560.33	2.90	0.98
28		16,727	5,100	511	1,270.00	3.90	0.95
29		19,108	5,100	524	878.26	3.90	0.96
30		22,013	5,100	244	549.04	3.20	0.98
31		62,690	5,100	373	719.24	3.84	0.97
32		50,771	5,100	420	836.33	3.82	0.96
33		76,535	5,100	488	1,034.86	4.48	0.95
34		115,711	5,100	445	843.40	4.12	0.96
35		151,898	5,100	930	1,900.68	5.52	0.91
36		124,883	5,100	793	2,393.73	4.43	0.90

37		53,348	5,100	635	1,329.69	4.64	0.94
38		35,361	5,100	547	1,446.33	4.94	0.94
39		35,443	5,100	413	1,101.29	4.60	0.96
40		24,283	5,100	468	1,143.28	4.30	0.96
41		24,048	5,100	1,160	3,956.75	5.64	0.84
42		53,564	5,100	722	1,929.25	4.24	0.91
43		55,576	5,100	982	2,941.14	5.24	0.87
44		26,396	5,100	664	1,370.12	4.31	0.93
45		41,459	5,100	633	1,240.45	4.18	0.93
46		17,327	5,100	351	751.12	3.38	0.96
47		33,295	5,100	675	1,393.33	4.45	0.93
1	Tumor tissue	25,691	5,100	492	999.88	3.77	0.95
2		17,373	5,100	335	732.50	4.54	0.97
3		13,372	5,100	635	1,241.94	5.03	0.94
4		5,199	5,100	183	426.67	2.50	0.98
5		12,697	5,100	441	810.75	4.03	0.96
6		28,394	5,100	343	665.35	4.22	0.97
7		15,548	5,100	338	669.53	4.55	0.97
8		30,172	5,100	301	607.08	4.18	0.97
9		16,325	5,100	432	935.52	4.42	0.96
10		25,381	5,100	738	1,718.64	5.35	0.93
11		6,714	5,100	620	1,752.16	4.68	0.92
12		13,730	5,100	42	69.20	1.30	0.99
13		25,402	5,100	715	1,775.54	5.05	0.92
14		14,194	5,100	516	1,091.40	4.02	0.95
15		35,109	5,100	684	1,464.41	4.29	0.93
16		53,499	5,100	117	377.00	0.76	0.99
17		46,751	5,100	418	776.02	4.78	0.97
18		27,250	5,100	507	1,029.64	3.81	0.95
19		31,191	5,100	567	1,320.67	3.88	0.94
20		35,698	5,100	238	586.00	1.45	0.97
21		26,881	5,100	557	1,206.30	4.03	0.94
22		10,947	5,100	417	923.47	3.69	0.96
23		13,173	5,100	457	989.78	3.76	0.96
24		6,759	5,100	401	775.06	4.40	0.96
25		15,830	5,100	641	1,419.54	4.47	0.93
26		31,363	5,100	91	201.00	0.97	0.99
27		14,429	5,100	428	949.78	3.69	0.96
28		10,816	5,100	398	720.20	3.41	0.96
29		8,076	5,100	345	794.69	3.39	0.97

30		20,931	5,100	142	307.67	2.47	0.99
31		35,355	5,100	632	1,182.61	4.52	0.94
32		49,564	5,100	539	1,478.00	4.99	0.94
33		93,655	5,100	444	850.12	4.14	0.96
34		47,983	5,100	466	928.85	4.46	0.96
35		172,967	5,100	463	889.62	4.47	0.95
36		117,721	5,100	491	1,215.71	4.61	0.95
37		152,028	5,100	364	687.43	3.45	0.97
38		46,964	5,100	418	940.50	3.72	0.96
39		42,936	5,100	338	516.22	4.08	0.97
40		28,387	5,100	409	884.23	4.67	0.96
41		20,022	5,100	332	809.97	4.42	0.97
42		17,629	5,100	1,040	3,131.13	5.07	0.86
43		37,577	5,100	994	2,960.15	5.35	0.87
44		68,363	5,100	1,441	5,666.12	5.85	0.80
45		42,123	5,100	1,473	5,183.50	5.53	0.79
46		40,043	5,100	632	1,262.92	4.34	0.94
47		13,059	5,100	410	759.52	3.63	0.96
1	Lymph node tissue	42,898	5,100	609	1,170.17	4.87	0.95
2		5,532	5,100	220	389.75	2.84	0.98
3		14,020	5,100	408	948.37	4.05	0.96
4		24,788	5,100	259	453.23	3.73	0.98
5		25,212	5,100	460	926.00	4.80	0.95
6		7,970	5,100	287	553.70	4.36	0.98
7		24,628	5,100	305	575.19	3.98	0.97
8		17,428	5,100	390	975.29	4.49	0.96
9		15,590	5,100	541	1,640.60	4.84	0.94
10		10,441	5,100	583	1,843.28	4.21	0.93
11		21,562	5,100	854	2,539.94	5.21	0.89
12		6,202	5,100	112	259.50	1.74	0.99
13		7,863	5,100	652	1,759.02	4.74	0.93
14		5,978	5,100	320	551.12	3.23	0.97
15		63,816	5,100	970	1,649.57	4.94	0.91
16		55,863	5,100	3	4.00	0.00	0.99
17		24,860	5,100	635	1,293.12	4.87	0.94
18		30,773	5,100	658	1,570.90	4.40	0.93
19		18,917	5,100	1,403	4,619.86	5.86	0.81
20		21,517	5,100	471	1,067.72	3.63	0.95
21		14,676	5,100	548	1,274.24	4.04	0.94
22		22,406	5,100	716	1,805.20	4.59	0.92

23		25,461	5,100	657	1,427.80	4.53	0.94
24		27,275	5,100	370	774.44	2.78	0.96
25		18,027	5,100	652	1,551.09	4.52	0.93
26		10,985	5,100	44	107.25	0.46	0.99
27		9,928	5,100	462	960.64	4.21	0.96
28		17,798	5,100	464	821.88	3.51	0.96
29		17,288	5,100	544	1,153.19	4.68	0.95
30		14,759	5,100	692	2,250.44	4.92	0.92
31		94,993	5,100	463	1,054.98	4.33	0.95
32		50,509	5,100	350	633.13	2.99	0.97
33		52,090	5,100	388	878.00	3.58	0.96
34		173,025	5,100	435	853.11	3.58	0.96
35		156,434	5,100	493	1,123.00	4.42	0.95
36		151,426	5,100	502	1,026.39	3.64	0.95
37		81,443	5,100	519	1,216.76	4.08	0.94
38		34,138	5,100	483	1,143.20	4.82	0.95
39		49,815	5,100	422	1,161.37	3.63	0.96
40		38,636	5,100	390	777.28	3.91	0.96
41		40,012	5,100	1,261	4,171.05	5.40	0.83
42		34,809	5,100	1,022	3,107.95	5.32	0.87
43		70,400	5,100	1,238	3,527.80	5.91	0.85
44		39,884	5,100	1,194	3,789.27	5.63	0.84
45		36,777	5,100	636	1,419.01	4.13	0.93
46		26,907	5,100	527	1,049.02	4.13	0.95
47		23,543	5,100	580	1,110.00	4.54	0.95

Table S2. Significantly different pathways between BCM1 and BCM2.

KEGG pathway category			Microbiota cluster (mean abundance ± SD)			
1st_category	2nd_category	3rd_category	BCM1	BCM2	*Q* value	Effect size
Metabolism	Carbohydrate Metabolism	Pentose and glucuronate interconversions	0.343 ± 0.050	0.448 ± 0.041	<0.0001	0.535
Metabolism	Glycan Biosynthesis and Metabolism	Other glycan degradation	0.051 ± 0.030	0.103 ± 0.018	<0.0001	0.477
Organismal Systems	Immune System	NOD-like receptor signaling pathway	0.027 ± 0.007	0.015 ± 0.003	<0.0001	0.468
Metabolism	Metabolism of Cofactors and Vitamins	Folate biosynthesis	0.465 ± 0.032	0.395 ± 0.045	0.0002	0.455
Genetic Information Processing	Folding, Sorting and Degradation	Sulfur relay system	0.370 ± 0.038	0.309 ± 0.019	0.0001	0.453
Organismal Systems	Endocrine System	Progesterone-mediated oocyte maturation	0.026 ± 0.007	0.015 ± 0.003	<0.0001	0.453
Organismal Systems	Immune System	Antigen processing and presentation	0.026 ± 0.007	0.015 ± 0.003	<0.0001	0.453
Metabolism	Xenobiotics Biodegradation and Metabolism	Polycyclic aromatic hydrocarbon degradation	0.112 ± 0.020	0.149 ± 0.019	0.0001	0.444
Metabolism	Carbohydrate metabolism	Carbohydrate metabolism_Unclassified	0.089 ± 0.029	0.139 ± 0.023	<0.0001	0.439
Metabolism	Glycan Biosynthesis and Metabolism	Glycosphingolipid biosynthesis - globo series	0.018 ± 0.014	0.042 ± 0.012	0.0001	0.433
Metabolism	Carbohydrate Metabolism	Galactose metabolism	0.343 ± 0.099	0.506 ± 0.089	<0.0001	0.402
